# *CDKN2B* downregulation and other genetic characteristics in T-acute lymphoblastic leukemia

**DOI:** 10.1038/s12276-018-0195-x

**Published:** 2019-01-11

**Authors:** Woori Jang, Joonhong Park, Ahlm Kwon, Hayoung Choi, Jiyeon Kim, Gun Dong Lee, Eunhee Han, Dong Wook Jekarl, Hyojin Chae, Kyungja Han, Jae-Ho Yoon, Seok Lee, Nack-Gyun Chung, Bin Cho, Myungshin Kim, Yonggoo Kim

**Affiliations:** 1 Department of Laboratory Medicine, College of Medicine, The Catholic University of Korea, Seoul, Korea; 2 Catholic Genetic Laboratory Center, College of Medicine, The Catholic University of Korea, Seoul, Korea; 3 Department of Hematology, Leukemia Research Institute, Catholic Hematology Hospital, Seoul St. Mary’s Hospital, College of Medicine, The Catholic University of Korea, Seoul, Korea; 4 Department of Pediatrics, College of Medicine, The Catholic University of Korea, Seoul, Korea

**Keywords:** Acute lymphocytic leukaemia, Cancer genetics

## Abstract

We identified principal genetic alterations in 97.1% (99/102) of patients with T-acute lymphoblastic leukemia (T-ALL) using integrative genetic analyses, including massive parallel sequencing and multiplex ligation-dependent probe amplification (MLPA). A total of 133 mutations were identified in the following genes in descending order: *NOTCH1* (66.7%), *FBXW7* (19.6%), *PHF6* (15.7%), *RUNX1* (12.7%), *NRAS* (10.8%), and *DNMT3A* (9.8%). Copy number alterations were most frequently detected in *CDKN2B*, *CDKN2A*, and genes on 9p21.3 in T-ALL (45.1%). Gene expression data demonstrated the downregulation of *CDKN2B* in most cases of T-ALL, whereas *CDKN2A* downregulation was mainly restricted to deletions. Additional quantitative methylation analysis demonstrated that *CDKN2B* downregulation stemmed from deletion and hypermethylation. Analysis of 64 patients with *CDKN2B* hypermethylation indicated an association with an older age of onset and early T cell precursor ALL, which involved very early arrest of T cell differentiation. Genes associated with methylation and myeloid neoplasms, including *DNMT3A* and *NRAS*, were more commonly mutated in T-ALL with *CDKN2B* hypermethylation. In particular, a *CDKN2B* biallelic deletion or high methylation level (≥45%), the age of onset, and the *GATA3* and *SH2B3* mutations were factors associated with a poor prognosis. This study clarifies that one of the most important genetic events in T-ALL, namely, *CDKN2B* downregulation, occurs mechanistically via deletion and hypermethylation. Different susceptible genetic backgrounds exist based on the *CDKN2B* downregulation mechanism.

## Acute leukemia: Key genetic characteristics uncovered

Crucial genetic events contributing to an aggressive form of leukemia have been identified by researchers in South Korea. T-acute lymphoblastic leukemia (T-ALL) is more common in adults than in children and accounts for around 20% of all leukemia cases. Myungshin Kim and Yonggoo Kim and co-workers at the Catholic University of Korea, Seoul conducted detailed genetic analysis of samples from 102 T-ALL patients aged 2–77 years and compared them with healthy controls. They found that 133 mutations on 6 genes were linked with the disease and showed that the reduced expression of one gene, *CDKN2B*, occurs in most T-ALL patients. *CDKN2B* expression levels were affected either by deletion or by modification via high levels of methylation. Biallelic deletion or high levels of methylation of *CDKN2B* was associated with poor prognosis in T-ALL.

## Introduction

T-acute lymphoblastic leukemia (T-ALL) is an aggressive hematologic malignancy that accounts for approximately 20% of all cases of ALL. T-ALL tends to be more common in adults than in children^1,2^. Efforts to develop a differential diagnosis of various leukemias have focused on morphology, immunophenotype, and molecular and/or cytogenetic factors. The genetic approach is hindered by the lack of information on T-ALL. Large-scale sequencing studies of the T-ALL genome have identified driver mutations involved in the loss of transcription factors, epigenetic tumor suppressors, cell cycle inhibitors, gain of oncogenes, and chromosomal rearrangements that can result in fusion products^3–6^. There is broad consensus that an aberrantly activated NOTCH1 pathway due to *NOTCH1* and *FBXW7* gene mutations is predominant in both pediatric and adult T-ALL. *NOTCH1* and *FBXW7* mutations have been reported in approximately 60% and 8–30% of T-ALL patients, respectively^3,4^. Deletion and hypermethylation of promoter sequences of the 9p21 region, which includes the *CDKN2A* and *CDKN2B* genes, are present in >70% of T-ALL cases^3,7,8^. In addition, alterations in *PHF6* (20%), *PTEN* (20%), *WT1* (15%), *RUNX1* (10–20%), *LEF1* (10–15%), and *ETV6* (5–15%) genes are present at a slightly lower frequency^3–5^. However, the mutational impact of these genes on survival is controversial based on different reports, even in the most extensively investigated cases affecting the *NOTCH1* gene^1,3,4,9^. This knowledge is insufficient to enable the genetic classification of T-ALL.

A further hindrance is the paucity of data on the role of genetic changes on risk stratification and prognosis in T-ALL patients compared with B cell ALL patients. A study involving patients with early T cell precursor (ETP)-ALL reported one subset with unique biology that might represent a provisional entity with a characteristic immunophenotypic and genetic profile (myeloid-associated gene mutations, such as mutations in *DNMT3A*, *ETV6*, *FLT3*, *GATA3*, *IDH1*, *IDH2*, *JAK3*, *NRAS*/*KRAS*, and *RUNX1*); the prognostic significance of the profile remains debated^10–12^.

Nevertheless, understanding the disease pathogenesis and expected biological behavior of T-ALL on the basis of genetic profiles is essential for the successful treatment of T-ALL. Thus we explored the association of the pathogenesis and biology of T-ALL based on genetic aberrations by integrative genetic analyses using massive parallel sequencing and copy number analysis of T-ALL patients. The goal was to identify commonly altered genetic aberrations. The identified genetic alterations were combined with gene expression and methylation data to understand the underlying mechanisms associated with the biological behaviors of T-ALL, such as surface antigen expression, and their prognostic impact.

## Materials and methods

### Patients

The study included 102 T-ALL patients (69 males and 33 females comprising 42 children and 60 adults; median age, 22 years (range, 2–77 years)) diagnosed between October 2004 and August 2015. The diagnosis of T-ALL was based on morphology, cytochemistry, and immunophenotyping characteristics according to the 2008 World Health Organization classifications. The median follow-up was 20.8 months from initial presentation (range, 0.5–134.7 months). Cytogenetic G-banding analysis was performed by standard methods. We also examined 28 leukemic fusion transcripts using a HemaVision multiplex RT-PCR system (DNA Diagnostic, Risskov, Denmark). ETP-ALL was defined based on the immunophenotype [CD1a(−), CD8(−) and CD5(−/dim)], stem cell expression phenotype (CD34, HLA-DR, CD117), and/or coexpression of myeloid antigens (CD13, CD33, CD11b, CD65)^13^. All patients were treated with remission induction chemotherapy according to our institutional protocol. Allogeneic hematopoietic stem cell transplantation (HSCT) was performed after consolidation. Autologous HSCT is used when an allogeneic donor is not available. Details of the treatment protocols were previously described^14–16^. Following the Declaration of Helsinki, all patients provided written informed consent for genetic analyses. The study protocol was approved by the Institutional Review Board of Seoul St. Mary’s Hospital, The Catholic University of Korea.

### Targeted capture and massive parallel sequencing of commonly mutated genes in T-ALL

To comprehensively characterize the patterns of somatic mutations in T-ALL, massive parallel sequencing of all coding exons of 11 genes (*NOTCH1*, *DNMT3A*, *FBXW7*, *RUNX1*, *PHF6*, *PTEN*, *GATA3*, *KRAS*, *EZH*, *NRAS*, and *SH2B3*) previously found to be associated with T-ALL was performed using semiconductor sequencing technology (IonTorrent PGM, Thermo Fisher Scientific, Carlsbad, CA, USA). Custom primers were designed online using the Ion AmpliSeq™ designer software (http://www.ampliseq.com; Thermo Fisher Scientific) to generate a total of 178 primer pairs in a two-pool design with predicted target coverage of 100%. Libraries were templated and enriched using Ion OneTouch™ 2 system and Ion OneTouch™ ES system (Thermo Fisher Scientific), respectively. Sequencing was performed on an Ion 318™ chip (Thermo Fisher Scientific) using the Sequencing Kit 200 (Thermo Fisher Scientific) per the manufacturer’s instructions.

Sequence alignment to the reference human genome (GRCh37/hg19) and base calling were performed using the Torrent Suite software version 4.2 (Thermo Fisher Scientific). Torrent Variant Caller v.4.2-r88446 (Thermo Fisher Scientific) was used for calling variants from PGM mapped reads, and the called variants were annotated using the Ion Reporter software version 4.4 (Thermo Fisher Scientific). Integrative Genomics Viewer facilitated the identification of variants. To minimize the risk of false positives, we required “novel” mutations to be detected at >10% of reads. Variants with a quality score >20, sequencing read depth >100, and variant allele frequency >4% were selected. Variants reported in dbSNP version 138, the 1000 Genomes Project, or the Korean Reference Genome databases and scored as tolerated or benign by Sorting Intolerant From Tolerant or polymorphism phenotyping methods were excluded. Variants were described according to the recommendations of the Human Genome Variation Society. Variant descriptions were assessed by Mutalyzer Name Checker (http://mutalyzer.nl). The mutant allele burden was calculated as the ratio of mutant reads to total reads for the affected base.

### Multiplex ligation-dependent probe amplification (MLPA) for the detection of commonly deleted genes in T-ALL

MLPA analysis was performed to detect commonly deleted and/or amplified genes in T-ALL using a SALSA MLPA Kit (P383-A1 T-ALL; MRC Holland, Amsterdam, The Netherlands) according to the manufacturer’s protocol. Amplification products were quantified and identified by capillary electrophoresis on an ABI 3130 Genetic Analyzer (Applied Biosystems, Foster City, CA, USA). Data were analyzed using the GeneMarker software v1.91 (SoftGenetics, State College, PA, USA). Intensity ratios of 0.6–1.4, 0.3–0.6, and 0.3 were considered to represent normal copy number (wild type), monoallelic deletion, and biallelic deletion, respectively.

### Measurement of *CDKN2A* and *CDKN2B* gene expression

To quantify the *CDKN2A* and *CDKN2B* mRNA expression, reverse transcription–quantitative polymerase chain reaction (RT-qPCR) was performed using the TaqMan® gene expression assay (Applied Biosystems) according to the manufacturer’s instructions. RNA was isolated from bone marrow (BM) aspirates of 49 patients and 6 normal controls using the High Pure RNA Isolation Kit (Roche Diagnostics, Mannheim, Germany). Taqman probes for the *CDKN2A* gene (HS00923894_m1) with transcript p16, *CDKN2B* gene (HS00793225_m1) with transcript p15, and the endogenous control (glyceraldehyde-3-phosphate dehydrogenase (*GAPDH*); Hs99999905_m1) were used. All reactions were performed using an ABI 7500 Real-Time PCR system (Applied Biosystems). Gene expression levels were calculated based on the 2^−ΔΔCt^ × 1000 method after normalization to the transcript levels of the *GAPDH* housekeeping gene.

### *CDKN2A* and *CDKN2B* promoter methylation analysis

CpG methylation in the promoter regions of the *CDKN2A* and *CDKN2B* genes was quantified using pyrosequencing. Bisulfite conversion of genomic DNA was performed using an Epitect Bisulfite Kit (Qiagen). Pyrosequencing was performed using the PyroMark® Gold Q96 Reagent Kit (Qiagen) according to the manufacturer’s instructions. All reactions were performed using the PyroMark^TM^ Q96 ID (Biotage AB, Uppsala, Sweden). A total of 12 CpG sites (*CDKN2A*, 5 sites; *CDKN2B*, 7 sites) were analyzed per sample. For *CDKN2A*, all assays were performed with commercially available gene-specific PyroMark CpG assays (Qiagen). The primers for the analysis of *CDKN2B*-specific CpG regions were designed using the Pyrosequencing Assay Design Software (Biotage AB, Supplementary Table S1).

### Statistical analyses

Differences in clinical variables according to mutation status were investigated using Fisher’s exact test for categorical variables and Mann–Whitney *U* test for continuous variables. Correlations were measured by Spearman’s Rho (*ρ*). Survival functions were calculated using a Kaplan–Meier survival analysis, and the differences in survival curves were compared using two-sided log-rank test. Cox proportional hazard models were used to estimate hazard ratios (HRs) for univariate and multivariate analyses for differences in survival. All statistical analyses were performed using SPSS 12.0.1 for Windows (SPSS, Chicago, IL, USA). A two-sided *P* value <0.05 indicated statistical significance.

## Results

### Genetic profile of T-ALL

We performed targeted NGS of 11 genes that were recurrently mutated in T-ALL. This gene panel includes *NOTCH1*^3,4^*, DNMT3A*^8,17^, *FBXW7*^3,4^, *RUNX1*^3,4,8,18^, *PHF6*^3,4^, *PTEN*^3,4^, *GATA3*^18^, *KRAS*^18^, *EZH2*^3,4,18^, *NRAS*^3,4^, and *SH2B3*^18^. These genes are mutated in T-ALL at relatively high frequencies, and six genes (*DNMT3A*, *RUNX1*, *GATA3*, *KRAS*, *EZH2*, and *SH2B3*) are associated with ETP-ALL based on previous reports.

**Fig. 1:**
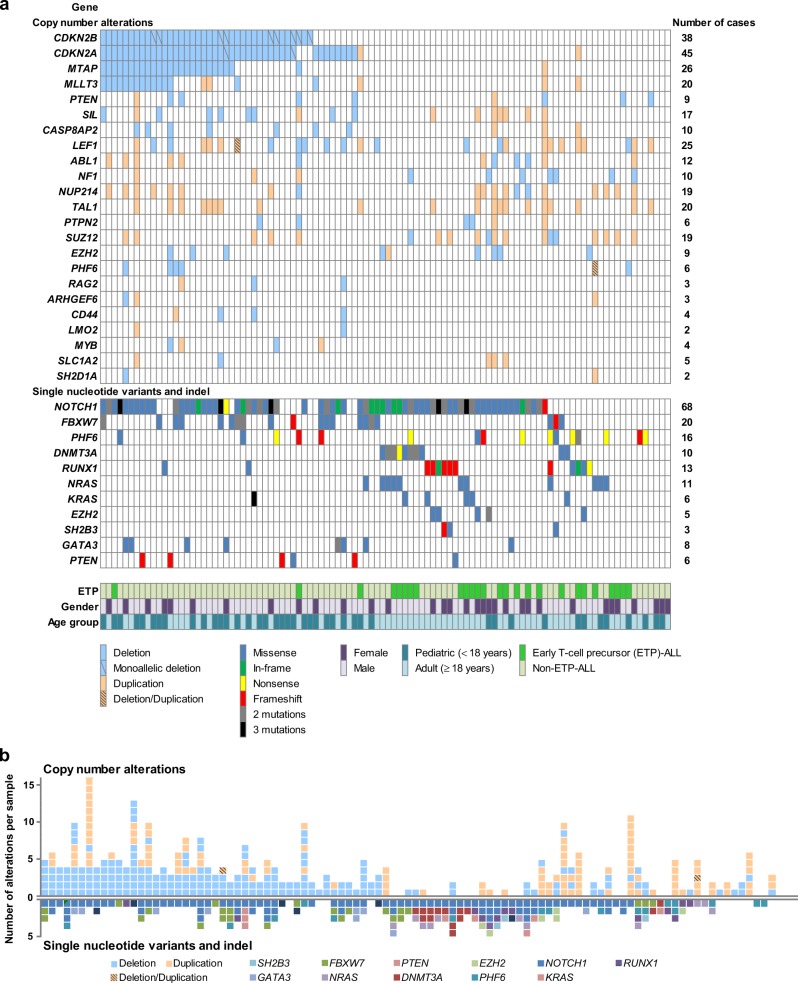
Overview of the genetic alterations identified in 102 T-ALL patients. **a** Single-nucleotide variations, small indels, and copy number alterations are presented for the 31 most frequently altered genes. Cases have been grouped by the presence or absence of a *CDKN2B* deletion, followed by cases with copy number alterations of genes on 9p21.3 (*CDKN2A*, *MTAP*, and *MLLT3* genes). The patient cohort is further annotated according to the ETP status, gender, and age groups. Each column represents one patient, and each colored box indicates a mutation. **b** Each colored box represents each genetic alteration (copy number alterations, single-nucleotide variations, and small indels) per gene. Each column corresponds to one sample

Among all enrolled T-ALL patients, 97.1% (99/102) possessed at least one somatic mutation and/or copy number alteration (Fig. 1a). A total of 133 non-synonymous somatic mutations were identified in 89 patients (87.3%). The number of mutations in each patient varied, ranging from 1 to 5 (median, 2). The majority of the mutations detected were missense mutations (76/133, 57.1%), followed by frameshift (25/133, 18.8%), nonsense (18/133, 13.5%), and in-frame (14/133, 10.5%) mutations. The median mutant allele burden was 40.0% (range, 3.5–98.4%).

**Fig. 2:**
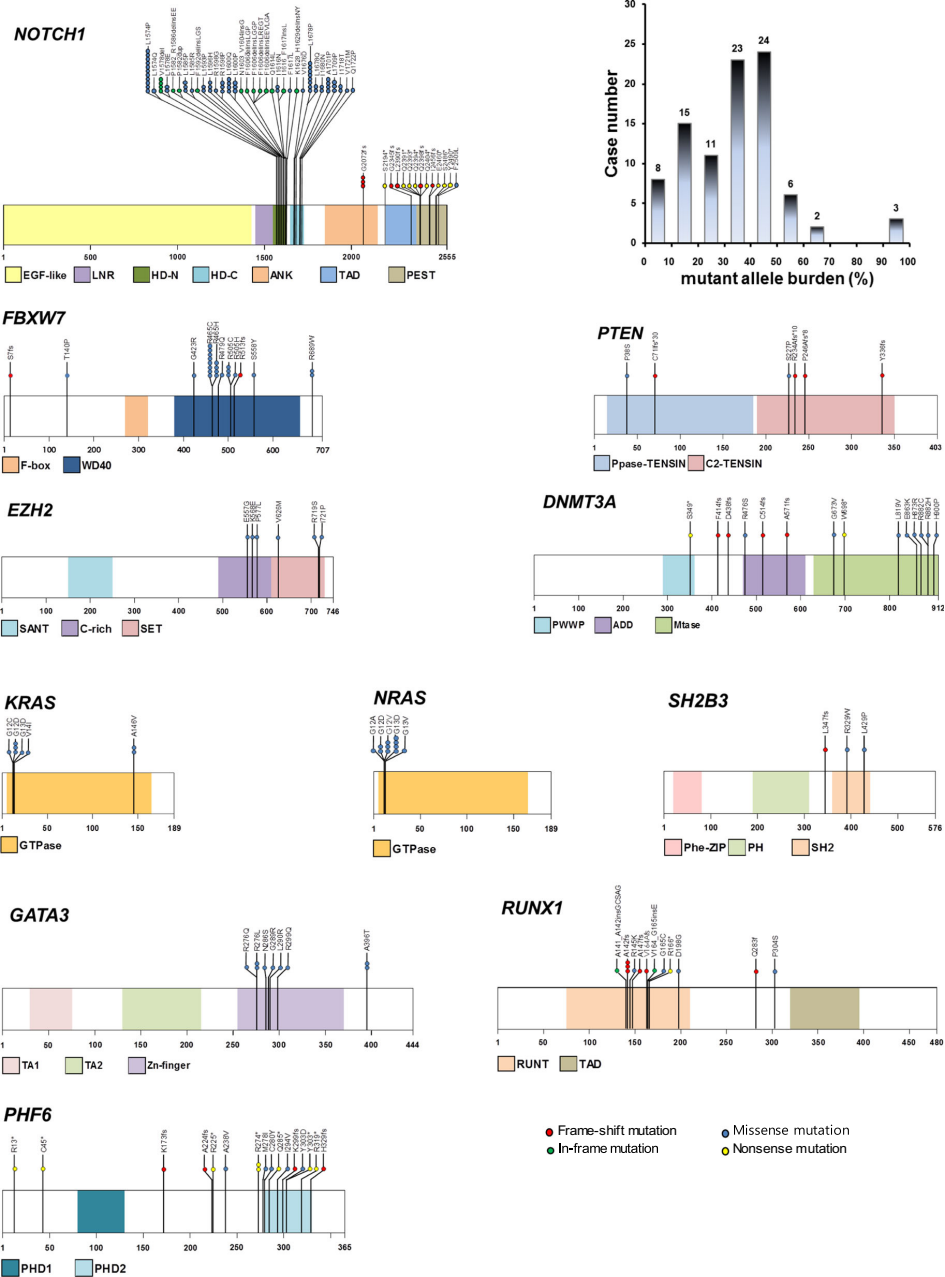
Sequence mutations for 11 molecular markers in our T-ALL cohort. The frequency of the obtained mutations is illustrated by the number of colored circles, which also indicate the effect at the amino acid level. The histogram presents the distribution of the *NOTCH1* mutant allele level detected in our T-ALL cases. The schematics are based on the following NCBI protein reference sequences: *NOTCH1*, NP_060087.3; *FBXW7*, NP_361014.1; *PTEN*, NP_000305.3; *EZH2*, NP_004447.2; *DNMT3A*, NP_072046.2; *KRAS*, NP_ 203524.1; *NRAS*, NP_002515.1; *SH2B3*, NP_005466.1; *GATA3*, NP_001002295.1; *RUNX1*, NP_001745.2; and *PHF6*, NP_115834.1

*NOTCH1* mutations were the most frequent pathogenetic events in T-ALL patients (68/102, 66.7%). The 48 types of mutations observed comprised 23 missense, 12 in-frame, 8 nonsense, and 5 frameshift mutations. The mutations most commonly occurred in the heterodimerization (HD) domain (34/48, 71%) followed by the proline–glutamate–serine–threonine-rich (PEST) domain (11/48, 23%). Of the 19 patients who had ≥2 *NOTCH1* mutations, 10 had mutations in both the PEST and HD domains. The mutant allele burden was widely distributed (range, 3.5–98.4%; median, 36.3%) with two peaks at 10–20% and 40–50% (Fig. 2).

The mutational incidence in the other genes was as follows: *FBXW7* (20/102, 19.6%), *PHF6* (*n* = 16, 15.7%), *RUNX1* (*n* = 13, 12.7%), *NRAS* (*n* = 11, 10.8%), *DNMT3A* (*n* = 10, 9.8%), *GATA3* (*n* = 8, 7.8%), *PTEN* (*n* = 6, 5.9%), *KRAS* (*n* = 6, 5.9%), *EZH2* (*n* = 5, 4.9%), and *SH2B3* (*n* = 3, 2.9%). In total, 11 of the 16 *PHF6* mutations and 4 of the 6 *PTEN* mutations that were detected were frameshift or nonsense mutations, respectively, which could result in a loss-of-function mutation. By contrast, missense substitutions were predominantly observed in the other genes (Fig. 1a).

We used MLPA to identify copy number alterations in genes commonly altered in T-ALL^19,20^. The P383-A1 probe mix contains 56 probes for 13 different chromosomal regions: 1p33 (*SIL*-*TAL1*), 4q25 (*LEF1*), 6q15 (*CASP8AP2*), 6q23.3 (*MYB*), 7q36.1 (*EZH2*), 9p21.3 (*CDKN2A*, *CDKN2B*, *MTAP*, *MLLT3*), 9q34.1 (*NUP214*-*ABL1*), 10q23.31 (*PTEN*), 11p15.4 (*LMO1*), 11p13 (*LMO2*, *CD44*, *SLC1A2*, *RAG2*), 17q11.2 (*NF1*, *SUZ12*), 18p11.21 (*PTPN2*), and Xq26.2 (*SH2D1A*, *PHF6*, *ARHGEF6*). Copy number alterations were detected in 80/102 (78.4%) T-ALL patients and occurred in 23 genes. *CDKN2A* or *CDKN2B* deletions were observed in 46 of the 102 patients (45.1%). Among 43 patients with *CDKN2A* deletions, 41 (95.3%) harbored biallelic deletions. Monoallelic deletions were only detected in two patients. *CDKN2B* deletions were observed in 38 (37.3%) patients, including 76.3% biallelic (*n* = 29) and 23.7% monoallelic (*n* = 9) deletions. Both *CDKN2A* and *CDKN2B* deletions were noted in 35 (34.3%) of the T-ALL patients. Copy number alterations (deletion or gain) in other genes were as follows: *MTAP* (26/102, 25.5%), *LEF1* (*n* = 25, 24.5%), *MLLT3* (*n* = 20, 19.6%), and *TAL1* (*n* = 20, 19.6%). The *MTAP* and *MLLT3* genes on the 9p21.3 locus were co-deleted with *CDKN2A*/B in 25 and 20 patients, respectively. Interestingly, the number of copy number alterations in a patient was generally inversely correlated with the number of somatic mutations in a patient (Fig. 1b).

Some of the identified genetic lesions closely correlated with the age of onset. Copy number alterations in *CDKN2A* (66.7% vs. 28.3%, *P* < 0.001), *CDKN2B* (54.8% vs. 25.0%, *P* = 0.003), and *MLLT3* (33.3% vs. 10.0%, *P* = 0.005) were more prevalent among pediatric patients. *DNMT3A* and *KRAS* alterations were exclusively found in adults (*DNMT3A*, 16.7%, *P* = 0.005; *KRAS*, 10.0%, *P* = 0.041; Supplementary Figure S1).

### Characteristics of ETP-ALL

A total of 26 (25.5%) patients were classified as having ETP-ALL, which more commonly occurred in adult (≥18 years of age) patients (21/60, 35.0%) compared with pediatric (<18 years of age) patients (5/42, 11.9%) (*P* = 0.011). The characteristics of ETP-ALL are presented in Table 1. Some genetic profiles differed between ETP and non-ETP-ALL. *DNMT3A* mutations were more frequently detected in ETP-ALL (23.1% vs. 5.3%, *P* = 0.016), whereas deletions of the *CDKN2A*, *CDKN2B*, and *MTAP* genes located on 9p21 were more commonly noted in non-ETP-ALL (11.5% vs. 55.3%, 7.7% vs. 47.4%, and 7.7% vs. 31.6%, respectively; *P* < 0.001, *P* < 0.001, and *P* = 0.016, respectively). Three of the four *SET-NUP214* T-ALL were included in ETP-ALL (*P* = 0.036; Supplementary Figure S2).

**Table 1:** Characteristics and outcome of 102 T-ALL patients according to the ETP status

	Total	ETP subgroup		
		ETP (*n* = 26)	Non-ETP (*n* = 76)	*P*
Gender (M/F)	69/33	17/9	52/24	0.811
Age, years	21.5 (2–77)	44.5 (13–67)	18.0 (2–77)	<0.001
Laboratory findings at diagnosis				
Hb, g/dL	10.3 (3.9–17.5)	9.8 (4.4–16.3)	10.3 (3.9–17.5)	0.364
WBC, ×1000/μL	46.7 (0.7–676.6)	29.0 (2.0–402.2)	58.5 (0.7–676.6)	0.179
Platelets, ×1000/μL	65.0 (5.0–549.0)	84.5 (7.0–549.0)	61.0 (5.0–435.0)	0.034
PB blasts, %	76.0 (0.0–99.0)	76.5 (6.0–99.0)	75.5 (0.0–99.0)	0.616
BM blasts, %	93.0 (16.0–99.0)	91.0 (62.0–99.0)	93.3 (16.0–99.0)	0.914
*CDKN2B*				
Promoter methylation, %	50.6 (2.2–87.6)	73.2 (19.2–87.6)	30.6 (2.2–86.9)	0.001
Expression	0.2 (0.0–7.3)	0.3 (0.0–1.3)	0.2 (0.0–4.8)	0.341
Deletion, *n* (%)	38 (37.3)	2 (7.7)	36 (47.4)	<0.001
*CDKN2A*				
Promoter methylation, %	3.8 (0.5–44.4)	8.1 (1.6–24.2)	6.9 (0.5–44.4)	0.001
Expression	7.1 (0.02–238.9)	41.3 (12.0–238.9)	0.7 (0.02–199.9)	<0.001
Deletion, *n* (%)	43 (42.2)	1 (3.8)	42 (55.3)	<0.001
Mutation				
* NOTCH1*				1.000
Negative, *n* (%)	34 (33.3)	9 (34.6)	25 (32.9)	
Positive, *n* (%)	68 (66.7)	17 (65.4)	51 (67.1)	
* DNMT3A*				0.016
Negative, *n* (%)	92 (90.2)	20 (76.9)	72 (94.7)	
Positive, *n* (%)	10 (9.8)	6 (23.1)	4 (5.3)	
* FBXW7*				0.091
Negative, *n* (%)	82 (80.4)	24 (92.3)	58 (76.3)	
Positive, *n* (%)	20 (19.6)	2 (7.7)	18 (23.7)	
* RUNX1*				0.507
Negative, *n* (%)	89 (87.3)	24 (92.3)	65 (85.5)	
Positive, *n* (%)	13 (12.7)	2 (7.7)	11 (14.5)	
* PHF6*				0.546
Negative, *n* (%)	86 (84.3)	21 (80.8)	65 (85.5)	
Positive, *n* (%)	16 (15.7)	5 (19.2)	11 (14.5)	
* PTEN*				0.334
Negative, *n* (%)	96 (94.1)	26 (100)	70 (92.1)	
Positive, *n* (%)	6 (5.9)	0 (0)	6 (7.9)	
* GATA3*				0.110
Negative, *n* (%)	94 (92.2)	26 (100)	68 (89.5)	
Positive, *n* (%)	8 (7.8)	0 (0)	8 (10.5)	
* KRAS*				0.171
Negative, *n* (%)	96 (94.1)	23 (88.5)	73 (96.1)	
Positive, *n* (%)	6 (5.9)	3 (11.5)	3 (3.9)	
* EZH2*				0.600
Negative, *n* (%)	97 (95.1)	24 (92.3)	73 (96.1)	
Positive, *n* (%)	5 (4.9)	2 (7.7)	3 (3.9)	
* NRAS*				0.142
Negative, *n* (%)	91 (89.2)	21 (80.8)	70 (92.1)	
Positive, *n* (%)	11 (10.8)	5 (19.2)	6 (7.9)	
* SH2B3*				0.568
Negative, *n* (%)	99 (97.1)	26 (100)	73 (96.1)	
Positive, *n* (%)	3 (2.9)	0 (0)	3 (3.9)	
CR rate (%)	83 (81.4)	22 (84.6)	61 (80.3)	0.774
Relapse (%)	33 (32.4)	6 (23.1)	27 (35.5)	0.333
Death (%)	48 (47.1)	10 (38.5)	38 (50.0)	0.367

Data are presented as median (range) unless otherwise indicated

*T-ALL* T-acute lymphoblastic leukemia, *ETP* early T- cell precursor, *M* male, *F* female, *Hb* hemoglobin, *WBC* white blood cell, *PB* peripheral blood, *BM* bone marrow, *CR* complete remission

### Downregulation of *CDKN2B* gene expression in T-ALL

**Fig. 3:**
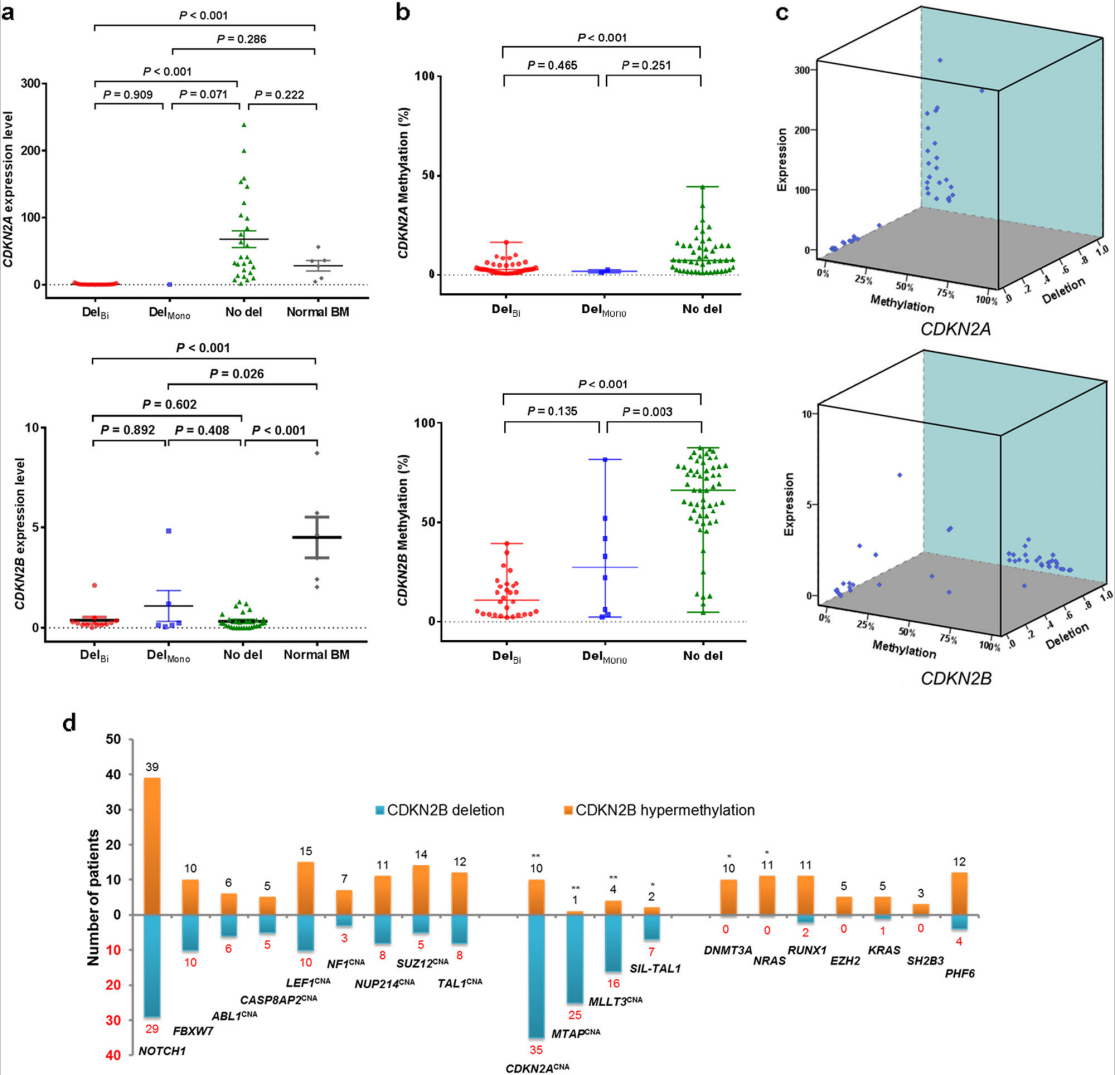
Copy number alterations, promoter methylation, and expression of *CDKN2A* and *CDKN2B* genes in T-ALL. **a** Expression level of *CDKN2A* and *CDKN2B* genes in T-ALL cases and normal bone marrow. Comparison among biallelic deletion (Del_Bi_), monoallelic deletion (Del_Mono_), no deletion (No del), and normal bone marrow. **b** Comparison of methylation levels of *CDKN2A* and *CDKN2B* genes between groups in T-ALL cases. **c** Three-dimensional (3D) scatter plot of the relationships among gene expression level, methylation level, and gene deletion. *CDKN2A* downregulation is mainly confined to the cases with *CDKN2A* deletion. *CDKN2B* gene downregulation is observed in almost all cases of T-ALL. *CDKN2B* deletion and hypermethylation appear to be mutually exclusive in our cohort. **d** Frequency and distribution of recurring genetic alterations according to the *CDKN2B* status (deletion vs. hypermethylation). **P < 0.001; * *P* < 0.05; CNA copy number alteration

To understand whether these changes corresponded to changes in gene expression, *CDKN2A* and *CDKN2B* gene expression was measured by RT-qPCR in 49 available T-ALL samples, and the values were compared with the value attained from normal BM. *CDKN2A* expression did not differ between T-ALL and normal BM (median, 7.137; range, 0.048–238.943 vs. 31.523; 4.445–56.243, *P* = 0.307). Among those with T-ALL, patients with a *CDKN2A* deletion revealed significantly reduced *CDKN2A* expression compared with those without a deletion (0.258; 0.048–3.176 vs. 41.288; 1.912–238.943, *P* < 0.001; Fig. 3a, c). *CDKN2B* expression was significantly reduced in patients with T-ALL (median, 0.221; range, 0.000–4.833) compared with those with normal BM (median, 4.067; range, 2.043–8.721, *P* < 0.001). Of note, *CDKN2B* expression was reduced in patients with a *CDKN2B* deletion (median, 0.208; range, 0.010–4.833) and those without a deletion (median, 0.221; range, 0.000–1.303, *P* = 0.406) (Fig. 3a, c). The results indicate a downregulation of *CDKN2B* in most cases of T-ALL. However, *CDKN2A* downregulation is mainly restricted to cases with a *CDKN2A* deletion.

### *CDKN2B* hypermethylation in T-ALL

Given that promoter hypermethylation is an alternative method to inactivate tumor-suppressor genes in a variety of malignancies, we evaluated the methylation status of 93 *CDKN2A* and *CDKN2B* genes. The median methylation level of *CDKN2B* CpG sites was 50.6% (range, 2.2–87.6%), which was increased compared with *CDKN2A* (median, 3.8%; range, 0.5–44.4%, *P* < 0.001). The methylation levels of the *CDKN2B* and *CDKN2A* genes were positively correlated (Spearman rho = 0.55, *P* < 0.001). *CDKN2A* and *CDKN2B* methylation was dependent on the deletion status of each gene. Patients without a *CDKN2A* deletion exhibited increased *CDKN2A* methylation compared to those with a *CDKN2A* deletion (median, 7.1%; range, 1.1–44.4% vs. 2.6%; 0.5–16.3%, *P* < 0.001). *CDKN2B* methylation in patients without *CDKN2B* deletion was significantly increased compared with those with a deletion (median, 66.3%; range, 5.1–87.6% vs. 13.3%; 2.2–81.5%, *P* < 0.001). All patients without *CDKN2B* deletion exhibited a >5% *CDKN2B* methylation level. The methylation level was highest in patients without *CDKN2B* deletion, followed by monoallelic and biallelic deletions (median, 27.5%; range, 2.4–81.5% and 11.0%; 2.2–39.3%, respectively; Fig. 3b, c). These methylation and gene expression data indicate that hypermethylation is the main downregulation mechanism of *CDKN2B* expression, especially in patients without *CDKN2B* deletion.

### Comparison of *CDKN2B* deletion and hypermethylation

Based on the above results, we grouped patients according to the *CDKN2B* downregulation mechanism. As expected, no significant difference in *CDKN2B* expression was noted between the two groups: deletion and hypermethylation (median, 0.2; range, 0.01–4.8 vs. 0.2; 0.0–7.3, *P* = 0.436, respectively). The clinical characteristics and genetic profiles were influenced by the downregulation mechanism (Table 2). T-ALL with *CDKN2B* deletion was diagnosed at a younger age than in patients harboring hypermethylation (median, 15.7 vs. 29.5 years, *P* = 0.001) and was associated with lower platelet counts (median, 52.0 × 1000/μL vs. 79.0 × 1000/μL, *P* = 0.018) and higher BM blast values (median, 95.0% vs. 91.0%, *P* = 0.043). Interestingly, most of the ETP-ALL cases (92.3%) were included in the *CDKN2B* hypermethylation group. *DNMT3A* (*P* = 0.012) and *NRAS* (*P* = 0.006) mutations exclusively occurred in the *CDKN2B* hypermethylation group. *RUNX1*, *EZH2*, *KRAS*, *SH2B3*, and *PHF6* mutations tended to occur more frequently in the *CDKN2B* hypermethylation group. However, the results did not reach statistical significance, perhaps due to the limited number of patients (Fig. 3d, Supplementary Figure S3 and S4).

**Table 2:** Comparison of characteristics and outcome between patients with *CDKN2B* deletion and *CDKN2B* hypermethylation

	*CDKN2B* subgroup		
	Deletion (n = 38)	Hypermethylation (n = 64)	*P*
Gender (M/F)	29/9	40/24	0.191
Age, years	15.7 (7–58)	29.5 (2–77)	0.001
Laboratory findings at diagnosis			
Hb, g/dL	11.2 (6.1–15.9)	10.0 (3.9–17.5)	0.155
WBC, ×1000/μL	59.2 (1.2–676.6)	36.6 (0.7–577.0)	0.200
Platelets, ×1000/μL	52.0 (6.0–435.0)	79.0 (5.0–549.0)	0.018
PB blasts, %	76.0 (0.0–99.0)	74.5 (0.0–99.0)	0.961
BM blasts, %	95.0 (16.0–99.0)	91.0 (22.0–99.0)	0.043
*CDKN2B*			
Promoter methylation, %	13.3 (2.2–81.5)	66.3 (4.8–87.6)	<0.001
Expression	0.2 (0.01–4.8)	0.2 (0.0–7.3)	0.436
*CDKN2A*			
Promoter methylation, %	2.6 (0.5–10.0)	7.0 (0.6–44.4)	<0.001
Expression	0.4 (0.1–32.4)	31.5 (0.05–238.9)	<0.001
Deletion, *n* (%)	35 (92.1)	8 (12.5)	<0.001
Mutation			
* NOTCH1*			0.132
Negative, *n* (%)	9 (23.7)	25 (39.1)	
Positive, *n* (%)	29 (76.3)	39 (60.9)	
* DNMT3A*			0.012
Negative, *n* (%)	38 (100)	54 (84.4)	
Positive, *n* (%)	0 (0)	10 (15.6)	
* FBXW7*			0.206
Negative, *n* (%)	28 (73.7)	54 (84.4)	
Positive, *n* (%)	10 (26.3)	10 (15.6)	
* RUNX1*			0.124
Negative, *n* (%)	36 (94.7)	53 (82.8)	
Positive, *n* (%)	2 (5.3)	11 (17.2)	
* PHF6*			0.400
Negative, *n* (%)	34 (89.5)	52 (81.3)	
Positive, *n* (%)	4 (10.5)	12 (18.8)	
* PTEN*			0.192
Negative, *n* (%)	34 (89.5)	62 (96.9)	
Positive, *n* (%)	4 (10.5)	2 (3.1)	
* GATA3*			0.466
Negative, *n* (%)	34 (89.5)	60 (93.8)	
Positive, *n* (%)	4 (10.5)	4 (6.3)	
* KRAS*			0.407
Negative, *n* (%)	37 (97.4)	59 (92.2)	
Positive, *n* (%)	1 (2.6)	5 (7.8)	
* EZH2*			0.154
Negative, *n* (%)	38 (100)	59 (93.8)	
Positive, *n* (%)	0 (0)	5 (6.3)	
* NRAS*			0.006
Negative, *n* (%)	38 (100)	53 (82.8)	
Positive, *n* (%)	0 (0)	11 (17.2)	
* SH2B3*			0.292
Negative, *n* (%)	38 (100)	61 (95.3)	
Positive, *n* (%)	0 (0)	3 (4.7)	
CR rate (%)	35 (92.1)	48 (75.0)	0.037
Relapse (%)	15 (39.5)	18 (28.1)	0.167
Death (%)	15 (39.5)	33 (51.6)	0.164
ETP (%)	2 (5.3)	24 (37.5)	<0.001

Data are presented as median (range) unless otherwise indicated

*M* male, *F* female, *Hb* hemoglobin, *WBC* white blood cell, *PB* peripheral blood, *BM* bone marrow, *CR* complete remission, *ETP* early T cell precursor

### Prognosis

**Fig. 4:**
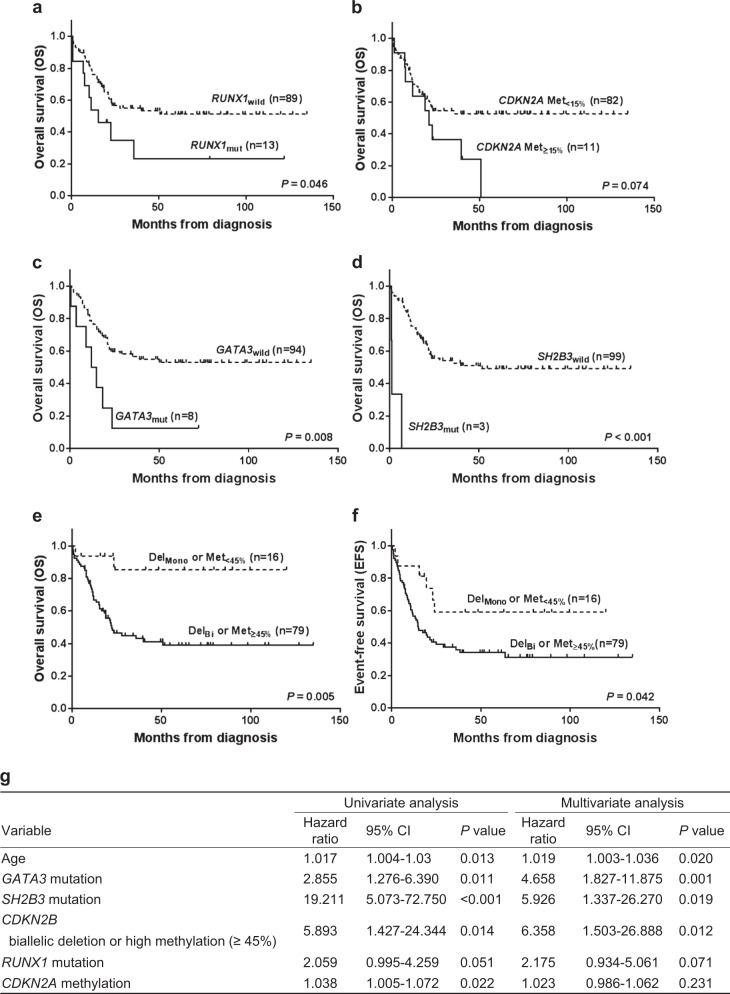
Prognostic factors for T-ALL patients. *RUNX1* mutation (**a**) and *CDKN2A* hypermethylation (**b**) were associated with reduced OS in T-ALL patients. Met_<15%_ methylation level <15%, Met_≥15%_ methylation level ≥15%. *GATA3* (**c**) and *SH2B3* (**d**) mutations were independent markers of a poor prognosis for the OS in T-ALL patients. Kaplan–Meier analyses for OS (**e**) and EFS (**f**) for patients with T-ALL according to *CDKN2B* status (deletion and methylation). Del_Mono_ monoallelic deletion, Del_Bi_ biallelic deletion, Met_<45%_ methylation level <45%, Met_≥45%_ methylation level ≥45%. **g** Univariate and multivariate analyses of factors associated with overall survival in T-ALL patients

To assess the clinical relevance of genetic alterations, their prognostic significance was examined in a cohort of 102 T-ALL cases. The estimated mean overall survival (OS) and event-free survival (EFS) were 72.1 months (95% confidence interval (CI), 59.5–84.7) and 58.9 months (46.7–71.0), respectively, among all patients. ETP-ALL did not exhibit different OS and EFS values compared with non-ETP-ALL in our cohort. Among the genetic alterations, *RUNX1* mutation (HR, 2.059; CI, 0.995–4.259; *P* = 0.051) and *CDKN2A* methylation (HR, 1.038; CI, 1.005–1.072; *P* = 0.022) were associated with reduced OS by univariate analysis. However, these factors did not reach statistical significance by multivariate analysis (Fig. 4a, b, g). Small cohorts of 8 and 3 patients with *GATA3* and *SH2B3* mutations, respectively, exhibited shorter OS compared with wild-type patients (*GATA3* mutated vs. wild-type: median OS, 13.4 months vs. not reached, *P* = 0.008; *SH2B3* mutated vs. wild-type: median OS, 1.2 months vs. 50.8 months, *P* < 0.001). *NOTCH1* mutations including the involved domain and mutant allele burden did not influence disease prognosis in our cohort. In multivariate analyses, age of onset (HR, 1.019; CI, 1.003–1.036; *P* = 0.020), *GATA3* mutation (HR, 4.658; CI, 1.827–11.875; *P* = 0.001), and *SH2B3* mutation (HR, 5.926; CI, 1.337–26.270; *P* = 0.019) were identified as independent markers of a poor prognosis (Fig. 4c, d, g). No significant differences in survival were noted between patients with and without *CDKN2A* gene deletion. No statistically significant difference in OS was observed between patients with *CDKN2B* gene deletion and hypermethylation.

In addition, we designed genetic indices predictive of a poor outcome based on the combination of *CDKN2B* methylation and deletion status, which are common mechanisms of downregulation. Patients with either biallelic deletion or high methylation level (≥45%) were considered to exhibit a poor prognosis given that their estimated 3-year OS and EFS were shorter than those with monoallelic deletion or low (<45%) methylation (OS: 43.0% vs. 85.2%, *P* = 0.005; EFS: 35.9% vs. 59.1%, *P* = 0.042). The majority of patients in the former group were more likely to die during follow-up (44/79, 55.7%) compared with the latter (2/16, 12.5%, *P* = 0.002). *CDKN2B* biallelic deletion or high methylation remained an important independent significant prognostic factor after multivariate analysis (HR, 6.358; CI, 1.503–26.888; *P* = 0.012; Fig. 4e–g). Given that age was a significant prognostic factor, survival analyses were conducted for each of the two age groups. *GATA3* and *SH2B3* mutations were associated with poor OS in adult patients (*P* = 0.008 and *P* < 0.001, respectively). *GATA3, SH2B3*, *NRAS*, and *FBXW7* mutations were associated with poor EFS in adult patients (*P* = 0.022, *P* = 0.001, *P* = 0.033, and *P* = 0.018). In pediatric patients, *CDKN2A* gene deletion was associated with an inferior EFS (*P* = 0.033).

## Discussion

The *CDKN2B* gene encoding the tumor-suppressor p15^INK4B^ is closely chromosomally linked to *CDKN2A* and is involved in the cell cycle and senescence. This gene is frequently inactivated by deletion, methylation, or mutation in a wide variety of tumors^21,22^. In acute leukemias, homozygous deletion of *CDKN2B* and *CDKN2A* occurs in approximately 30% of ALL patients^23^. In T-ALL, deletion occurred in approximately 50% of childhood T-ALL patients, whereas mutation is rarely observed^24^. Methylation studies demonstrated that the *CDKN2B* gene but not *CDKN2A* is frequently inactivated by hypermethylation of the promoter in acute leukemias^25–27^. However, few T-ALL cases were included in these studies, and the functional implications of *CDKN2B* in T-ALL pathogenesis and prognostic significance remain uncharacterized.

To our knowledge, this study is the first study to demonstrate that *CDKN2B* downregulation mechanisms are activated in most T-ALL cases. Reduced *CDKN2B* gene expression was clearly validated by RT-qPCR. One downregulation mechanism involves gene deletion. *CDKN2B* deletion was observed in 37.3% of our T-ALL cohort. Previous studies have demonstrated that *CDKN2B* and *CDKN2A* are inactivated by monoallelic or biallelic deletion in a number of ALL types^6,24,28,29^. In the current and prior studies^28,29^, most *CDKN2B* deletions (35/38, 92.1%) occurred along with *CDKN2A* deletion. *MTAP*, *MLLT3*, and *CDKN2A* genes were also frequently deleted with *CDKN2B*. Therefore, these codeletions are thought to result from the genetic instability of chromosome 9p21.3 in T-ALL. However, because the previous study design involved mixed B-ALL with T-ALL^23^, and the techniques used did not enable discrimination of the gene(s) that were actually deleted^28,30,31^, it is difficult to gauge the significance of individual gene deletion and to identify which factor contributes more to the pathogenesis and biological behavior. *CDKN2A* deletion is generally considered to arise as a secondary event cooperatively with initiating driver mutations^24,32^. Few studies have addressed *CKDN2B* downregulation in T-ALL. Reduced *CDKN2B* expression in individuals with the rs77728904 polymorphism has been reported to be associated with an increased risk of B cell precursor ALL^33^. We used MLPA to easily discriminate the deleted gene. *CDKN2A* gene expression was reduced along with the deletion, whereas *CDKN2B* gene expression was low, even in cases with no deletion. This consistent downregulation indicates that *CDKN2B* plays a more important role in T-ALL pathogenesis compared with *CDKN2A*.

The current results indicate that another *CDKN2B* downregulation mechanism is hypermethylation. Hypermethylation of the CpG island in promoter regions is critical for the regulation of gene expression. The comprehensive analysis accounts for different influences of two genes on T-ALL even though they are located close together. Unlike *CDKN2A*, *CDKN2B* was hypermethylated in >5% of patients without a *CDKN2B* deletion. It was of particular interest to compare the characteristics between the two mechanisms. We identified several differences between the hypermethylation and deletion groups. *CDKN2B* hypermethylation was related to older age and ETP-ALL. Genes associated with the pathogenesis of acute myeloid leukemia, such as *DNMT3A*, *NRAS*, *RUNX1*, *EZH2*, and *KRAS*, were more commonly mutated in the hypermethylation group. These distinctive genetic differences are consistent with the findings in ETP-ALL involving very early arrest in T cell differentiation, which is related to hematopoietic stem cells and myeloid progenitors^8,18,34,35^. *DNMT3A* mutations are frequently observed in hematological malignancies and occur in a comparable range of lymphoid and myeloid disorders. Furthermore, the mutations are common in individuals with clonal hematopoiesis associated with aging^36^. *DNMT3A* mutations lead to localized hypermethylation affecting tumor-suppressor genes, including *CDKN2B*^37^; inhibited hematopoietic stem cell differentiation; and obstructed differentiation and leukemic transformation^38^. Thus the current results support the view that *DNMT3A* mutation is one of the *CDKN2B* hypermethylation-susceptible conditions in T-ALL.

We used targeted gene sequencing given that it is an effective method to detect a large number of actionable mutations in driver genes in T-ALL. Non-synonymous somatic mutations were identified in 87.3% of T-ALL cases with a median of two mutations per patient. A total of 133 mutations in 11 genes were identified. The mutations most frequently found were in *NOTCH1*, *FBXW7*, *PHF6*, *RUNX1*, *NRAS*, *DNMT3A*, and *GATA3* (reported in descending order of frequency). Of these, *NOTCH1* mutations were detected in 66.7% of T-ALL patients; 27.9% of patients had >2 mutations. Although most mutations were clustered in the HD domain (69%) and PEST domain (22%), each mutation was unique. Massive parallel sequencing was used in the study; the approach detects individual mutations and allows for measurement of the mutant burden, which represents the percentage of leukemic cells that harbor a specific mutation. The *NOTCH1* mutant burden ranged from 3.5% to 98.4%, and 23/93 (24.7%) mutations revealed a burden of <20%. We investigated the impact of mutant burden of *NOTCH1* mutations on disease phenotype and prognosis. The finding was consistent with prior descriptions that some *NOTCH1* mutations at diagnosis were present in minor subclones and were lost at relapse, indicating that such mutations are secondary events^30^. Although we did not identify any different characteristics according to the mutant burden and the location and type of mutations, the knowledge gained about mutations using massive parallel sequencing cannot be underestimated. This and further collaborative efforts will help definitively characterize the impact of mutant burden in T-ALL and clarify the characteristics of *NOTCH1* target therapy that will enhance its success.

From the perspective of prognosis, age of onset and mutations in *RUNX1*, *GATA3*, and *SH2B3* were associated with poorer outcome. *CDKN2A* methylation also exhibited prognostic significance. Multivariate analyses identified age of onset, *GATA3* mutation, and *SH2B3* mutation as independent markers of a poor prognosis. A recent study revealed that *RUNX1* and *GATA3* were recurrent targets for mutation in ETP-ALL, and *GATA3* and *SH2B3* mutations were associated with poor prognosis in evaluating the relapse risk in T-ALL^18^. In another study, *GATA3* silencing occurred in approximately one third of adult ETP-ALL patients and was associated with *GATA3* DNA hypermethylation^39^. In addition, Perez-Garcia et al. reported that western blot analysis with a *SH2B3* C-terminal antibody revealed the complete loss of *SH2B3* protein expression in *SH2B3* mutant cells compared with cells derived from a *SH2B3* wild-type control^40^. Whether deletion or hypermethylation caused *CDKN2B* downregulation did not influence the clinical outcome. In most previous studies^4,41,42^, *CDKN2B* deletion did not play a prognostic role in T-ALL. Although it has been reported that hypermethylation of *CDKN2B* was associated with inferior EFS in ALL^27^, the prognostic impact of the methylation of *CDKN2B* on T-ALL remains inconclusive. This finding is a predictable result because the *CDKN2B* downregulation itself is important to T-ALL pathogenesis regardless of the mechanism of downregulation. However, the clinical outcome depended upon the indices affecting the level of downregulation. *CDKN2B* biallelic deletion or high methylation level (≥45%) was associated with a poorer outcome compared to monoallelic deletion or low methylation (<45%) (OS: 43.0% vs. 85.2%, *P* = 0.005). Particularly, the newly designed indices remained an important independent significant prognostic factor after multivariate analysis (HR, 6.358, *P* = 0.012).

This study identified principal genetic alterations in most of the T-ALL patients. In particular, we identified one of the most important genetic events in T-ALL, namely, *CDKN2B* downregulation, which results from two mechanisms: deletion and hypermethylation. Specific genetic backgrounds are susceptible to each mechanism, such as genetic instability on 9p21.3 and *DNMT3A* mutation, respectively. An integrative evaluation demonstrated that *CDKN2B* biallelic deletion or high methylation (≥45%) are factors associated with a poor prognosis, paving the way for trials of hypomethylating agents.

## Supplementary information

Supplementary Table S1

Supplementary Figure S1

Supplementary Figure S2

Supplementary Figure S3

Supplementary Figure S4
