# Supplementary material for: CDKN2B downregulation and other genetic characteristics in T-acute lymphoblastic leukemia
Source: Exp Mol Med. 2019 Jan 11;51(1):4. doi: 10.1038/s12276-018-0195-x (PMC6329696; doi:10.1038/s12276-018-0195-x)
Supplement: Supplementary file 1 — Supplementary Table S1 [file 12276_2018_195_MOESM1_ESM.doc]

**Supplementary Table S1.** Sequences of primers used for pyrosequencing analysis of *CDKN2B*-specific CpG regions

| PCR primer | | Pyrosequencing primer |
| --- | --- | --- |
| F | 5'-GTTGGTTTTTTATTTTGTTAGAG-3' | 5'-GGGGCTAGTGAGGATTT-3' |
| R | 5'-biotin-AACTCAACTTCATTACCCTCC-3' | 5'-TTTTTTAGAAGTAATGG-3' |
|  |  |  |
| F | 5'-GAGGGTAATGAAGTTGAGTTTAGGTTT-3' | 5'-TTTTAGGAAGGAGAGAGTG-3' |
| R | 5'-biotin-CCAAAAACTATCRCACCTTCTCCA-3' | 5'-GGTTAAYGGTGGATTATT-3' |
|  |  | 5'-ATGAGGGTTTGGTTAG-3' |
|  |  |  |
| F | 5'-GAGGGTAATGAAGTTGAGTTTAGGTTT-3' | 5'-GATAGTTTTTGGAAGT-3' |
| R | 5'-biotin-CCTAAAACCCCAACTACCTAAATC-3' |  |
|  |  |  |
| F | 5'-GGGATTAGTGGAGAAGGTG-3' | 5'-TGGGGTTTTAGGGTTT-3' |
| R | 5'-biotin-ACCAACRAAAACTCCTATACAAA-3' |  |

Abbreviations: F, forward; R, reverse.
