# Supplementary material for: CDKN2B downregulation and other genetic characteristics in T-acute lymphoblastic leukemia
Source: Exp Mol Med. 2019 Jan 11;51(1):4. doi: 10.1038/s12276-018-0195-x (PMC6329696; doi:10.1038/s12276-018-0195-x)
Supplement: Supplementary file 2 — Supplementary Figure S1 [file 12276_2018_195_MOESM2_ESM.pptx]

## Slide 1
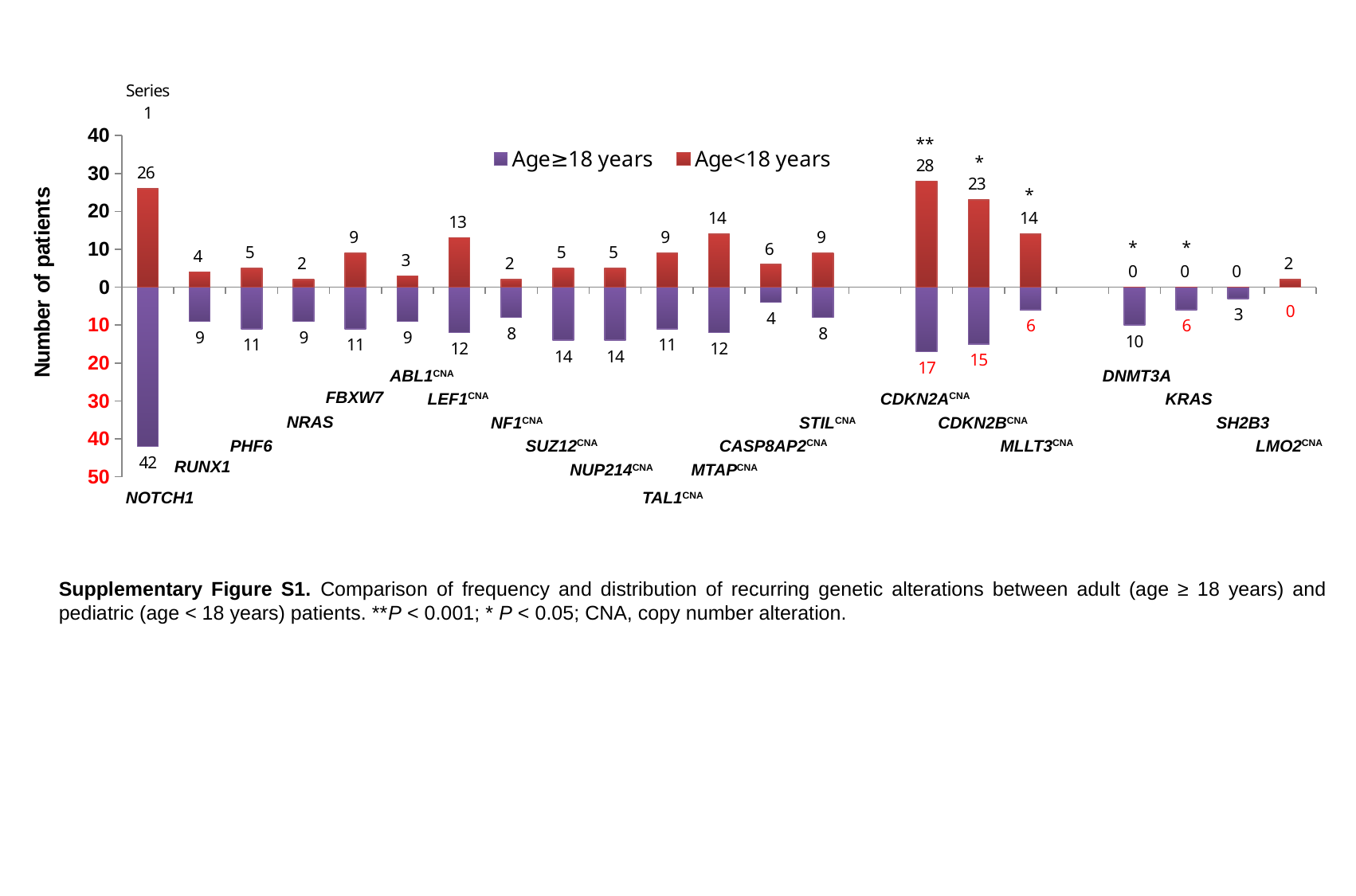

### Chart
| Category | Age≥18 years | Age<18 years |
|---|---|---|
| | -42.0 | 26.0 |
| | -9.0 | 4.0 |
| | -11.0 | 5.0 |
| | -9.0 | 2.0 |
| | -11.0 | 9.0 |
| | -9.0 | 3.0 |
| | -12.0 | 13.0 |
| | -8.0 | 2.0 |
| | -14.0 | 5.0 |
| | -14.0 | 5.0 |
| | -11.0 | 9.0 |
| | -12.0 | 14.0 |
| | -4.0 | 6.0 |
| | -8.0 | 9.0 |
| | None | None |
| | -17.0 | 28.0 |
| | -15.0 | 23.0 |
| | -6.0 | 14.0 |
| | None | None |
| | -10.0 | 0.0 |
| | -6.0 | 0.0 |
| | -3.0 | 0.0 |
| | 0.0 | 2.0 |**
*
*
*
*
ABL1CNA
DNMT3A
FBXW7
LEF1CNA
CDKN2ACNA
KRAS
NRAS
NF1CNA
STILCNA
CDKN2BCNA
SH2B3
SUZ12CNA
CASP8AP2CNA
MLLT3CNA
LMO2CNA
PHF6
RUNX1
NUP214CNA
MTAPCNA
NOTCH1
TAL1CNA
Supplementary Figure S1. Comparison of frequency and distribution of recurring genetic alterations between adult (age ≥ 18 years) and pediatric (age < 18 years) patients. **P < 0.001; * P < 0.05; CNA, copy number alteration.
