# Supplementary material for: CDKN2B downregulation and other genetic characteristics in T-acute lymphoblastic leukemia
Source: Exp Mol Med. 2019 Jan 11;51(1):4. doi: 10.1038/s12276-018-0195-x (PMC6329696; doi:10.1038/s12276-018-0195-x)
Supplement: Supplementary file 3 — Supplementary Figure S2 [file 12276_2018_195_MOESM3_ESM.pptx]

## Slide 1
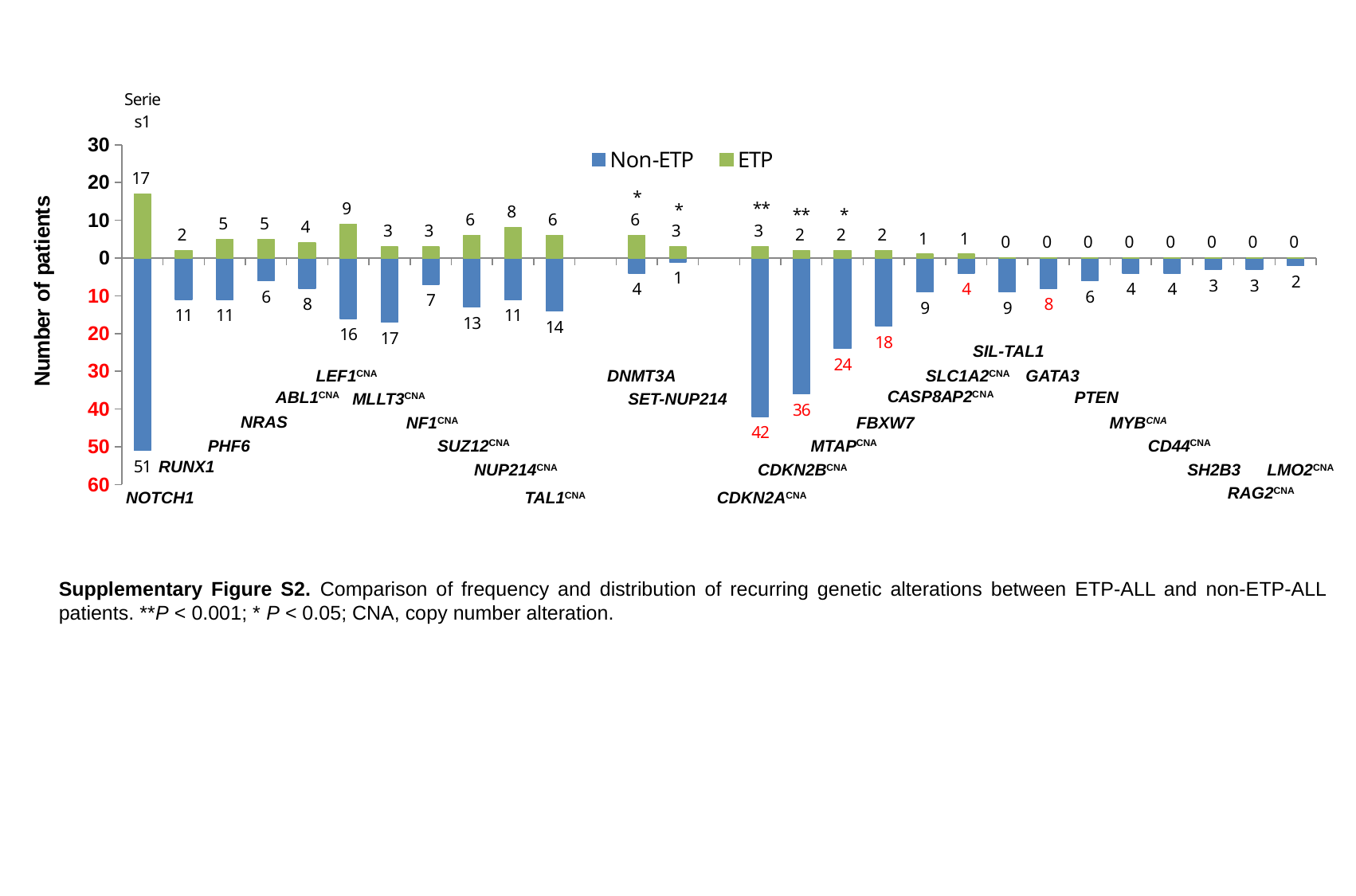

### Chart
| Category | Non-ETP | ETP |
|---|---|---|
| | -51.0 | 17.0 |
| | -11.0 | 2.0 |
| | -11.0 | 5.0 |
| | -6.0 | 5.0 |
| | -8.0 | 4.0 |
| | -16.0 | 9.0 |
| | -17.0 | 3.0 |
| | -7.0 | 3.0 |
| | -13.0 | 6.0 |
| | -11.0 | 8.0 |
| | -14.0 | 6.0 |
| | None | None |
| | -4.0 | 6.0 |
| | -1.0 | 3.0 |
| | None | None |
| | -42.0 | 3.0 |
| | -36.0 | 2.0 |
| | -24.0 | 2.0 |
| | -18.0 | 2.0 |
| | -9.0 | 1.0 |
| | -4.0 | 1.0 |
| | -9.0 | 0.0 |
| | -8.0 | 0.0 |
| | -6.0 | 0.0 |
| | -4.0 | 0.0 |
| | -4.0 | 0.0 |
| | -3.0 | 0.0 |
| | -3.0 | 0.0 |
| | -2.0 | 0.0 |SIL-TAL1
LEF1CNA
DNMT3A
SLC1A2CNA
GATA3
PTEN
ABL1CNA
MLLT3CNA
SET-NUP214
NRAS
NF1CNA
FBXW7
MYBCNA
SUZ12CNA
MTAPCNA
CD44CNA
PHF6
RUNX1
NUP214CNA
CDKN2BCNA
SH2B3
LMO2CNA
RAG2CNA
NOTCH1
TAL1CNA
CDKN2ACNA
Supplementary Figure S2. Comparison of frequency and distribution of recurring genetic alterations between ETP-ALL and non-ETP-ALL patients. **P < 0.001; * P < 0.05; CNA, copy number alteration.
