# Supplementary material for: CDKN2B downregulation and other genetic characteristics in T-acute lymphoblastic leukemia
Source: Exp Mol Med. 2019 Jan 11;51(1):4. doi: 10.1038/s12276-018-0195-x (PMC6329696; doi:10.1038/s12276-018-0195-x)
Supplement: Supplementary file 4 — Supplementary Figure S3 [file 12276_2018_195_MOESM4_ESM.pptx]

## Slide 1
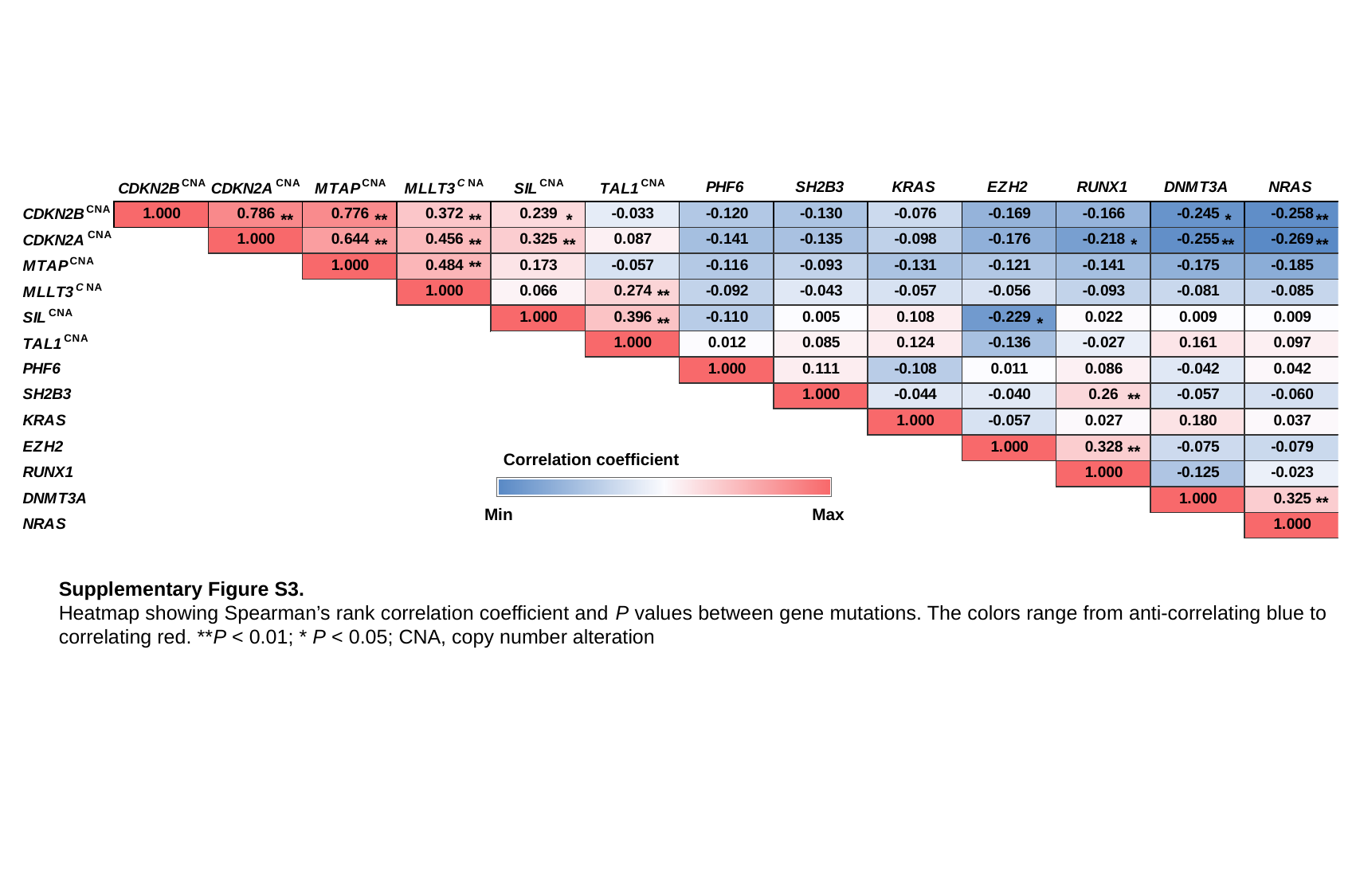

**
**
**
*
*
**
**
**
**
*
**
**
**
**
**
*
**
**
Correlation coefficient
**
Max
Min
Supplementary Figure S3.
Heatmap showing Spearman’s rank correlation coefficient and P values between gene mutations. The colors range from anti-correlating blue to correlating red. **P < 0.01; * P < 0.05; CNA, copy number alteration
