# Supplementary material for: CDKN2B downregulation and other genetic characteristics in T-acute lymphoblastic leukemia
Source: Exp Mol Med. 2019 Jan 11;51(1):4. doi: 10.1038/s12276-018-0195-x (PMC6329696; doi:10.1038/s12276-018-0195-x)
Supplement: Supplementary file 5 — Supplementary Figure S4 [file 12276_2018_195_MOESM5_ESM.pptx]

## Slide 1
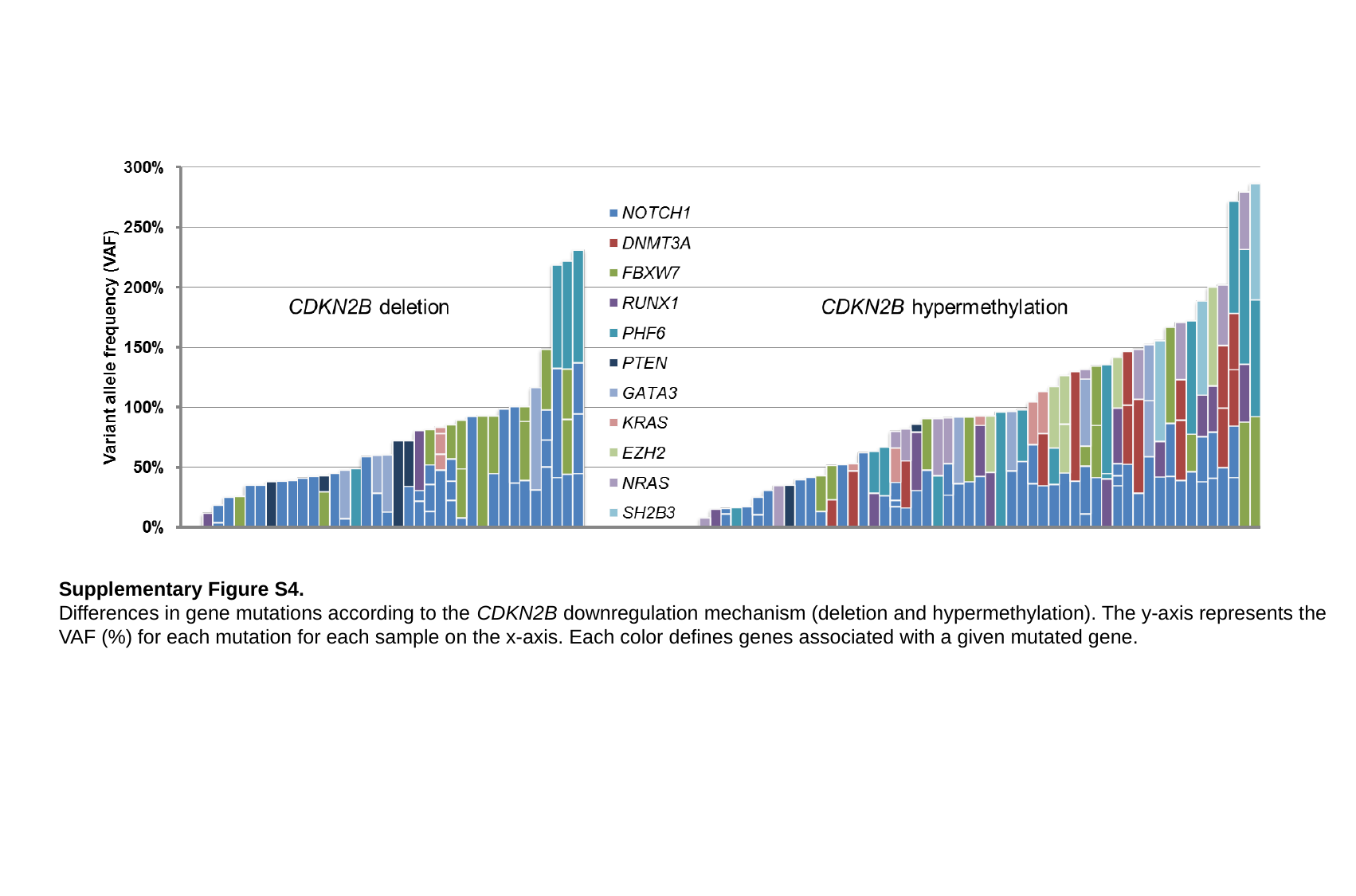

Supplementary Figure S4.
Differences in gene mutations according to the CDKN2B downregulation mechanism (deletion and hypermethylation). The y-axis represents the VAF (%) for each mutation for each sample on the x-axis. Each color defines genes associated with a given mutated gene.
